# Defining person-centred treatment support for multidrug-resistant TB: a discrete choice experiment

**DOI:** 10.5588/ijtldopen.25.0825

**Published:** 2026-06-15

**Authors:** M. Kagujje, G. Mtumbi, M. Sikandangwa, J. Shatalimi, M. Muyoyeta, A.D. Kerkhoff

**Affiliations:** 1 Centre for Infectious Disease Research in Zambia, Lusaka, Zambia;; 2 Division of HIV, Infectious Diseases, and Global Medicine, University of California San Francisco, San Francisco, CA, USA.

**Keywords:** tuberculosis, Zambia, drug resistant TB, drug treatment, personalised care, DCE

## Abstract

**BACKGROUND:**

Multidrug-resistant TB (MDR-TB) treatment remains challenging, with significant toxicity and associated hardships that undermine adherence and cure rates. Support packages may improve outcomes, but the features most valued by people with MDR-TB are unknown.

**METHODS:**

A discrete choice experiment was performed among adults receiving MDR-TB treatment in Lusaka, Zambia. Five features (3–4 levels each) comprising a support package were evaluated through 12 choice tasks comparing hypothetical packages.

**RESULTS:**

Among 99 participants (median age 36 years, 68.9% men, 42.4% HIV-positive), material support was the most valued feature (relative importance [RI] = 45.7%), with transport vouchers plus food assistance being the most preferred option. Visit frequency was also important (RI = 26.7%), with similar preferences for monthly and bimonthly visits. Participants preferred phone calls for visit reminders (RI = 11.8%), health care workers for emotional support (RI = 11.2%), and community-based health care workers or loved ones for treatment observation (RI = 4.7%). Three distinct preference groups were identified – all highly valued material support but varied in their preferences for other support features and their delivery.

**CONCLUSION:**

Among people with MDR-TB in Zambia, material support mechanisms and less frequent clinic visits were highly valued. Incorporating patient preferences into treatment programmes could optimise MDR-TB care and improve treatment adherence and outcomes.

Multidrug-resistant TB (MDR-TB) remains one of the most significant challenges to TB control globally. With an estimated 390,000 incident cases annually,^1^ MDR-TB poses substantial burdens for patients and health systems alike. While shorter, simplified regimens are increasingly available for MDR-TB, treatment remains demanding, with high daily pill burdens, significant toxicity, and considerable physical, emotional, and financial hardships for affected individuals.^2–4^ These challenges contribute to suboptimal treatment completion rates and unfavourable treatment outcomes, including death, particularly in high-burden, low-resource settings such as Zambia.^5,6^ Despite global treatment success rates for MDR-TB remaining unacceptably low at around 70%,^1^ the factors that undermine adherence and completion remain poorly addressed in many contexts. Many persons with MDR-TB face socio-economic vulnerabilities, including food insecurity, transportation challenges, and income loss, requiring comprehensive support beyond medication provision.^2–4^ Although the WHO recommends various patient support interventions (e.g., nutritional support, transportation vouchers, social support, and treatment support),^2^ if implemented, they are often based on health care system priorities rather than what people actually prefer or need.

There is increasing recognition that person-centred treatment support packages may enhance adherence and outcomes.^7–9^ In Zambia, where social and economic vulnerability is high among people with MDR-TB, understanding their preferences is particularly crucial for informing programmatic efforts to improve retention in care. Discrete choice experiments (DCEs) provide a methodologically robust approach for quantifying such preferences by requiring participants to make trade-offs between different package components, thereby identifying which elements matter most and helping prioritise limited resources for support packages that better align with patient needs and values.^10,11^

We conducted a DCE among adults receiving MDR-TB treatment in Lusaka to determine which features of a support package are most valued and to identify whether there are groups with distinct preferences. The findings can inform the development of more person-centred MDR-TB care delivery models in Zambia and similar settings.

## METHODS

We implemented a cross-sectional survey-based DCE at public health facilities in Lusaka, Zambia, among adults currently receiving MDR-TB treatment. Individuals ≥18 years old who had been on MDR/rifampicin-resistant treatment for at least 2 weeks and could provide informed consent were eligible to participate. Recruitment was conducted consecutively, with all eligible individuals approached during routine clinic visits at six MDR-TB treatment centres following Zambian national guidelines for MDR-TB management,^12^ and took place from October 2023 to February 2024. At the time of the study, MDR-TB care in Lusaka was decentralised to the zonal level. Patients could be initiated on a longer all-oral regimen (6–8 months bedaquiline [Bdq], linezolid [Lzd], levofloxacin [Lfx], clofazimine [Cfz]/12 months Lzd-Lfx-Cfz), a shorter regimen (4–6 months Bdq, Lfx/moxifloxacin [Mfx], Lzd, ethambutol [E], pyrazinamide [Z], high-dose isoniazid [Hh], Cfz/5 months Lfx[Mfx]-Cfz-Z-E), or an individualised regimen. Patients were reviewed every 2 weeks for the first month and, if clinically stable, transitioned to monthly reviews and refills thereafter. All patients were on directly observed therapy (DOT), with the majority monitored and supported by a relative. This study is reported in accordance with the Discrete Choice Experiment Reporting Checklist (DIRECT).^13^

### Design and procedures

**Figure 1.**
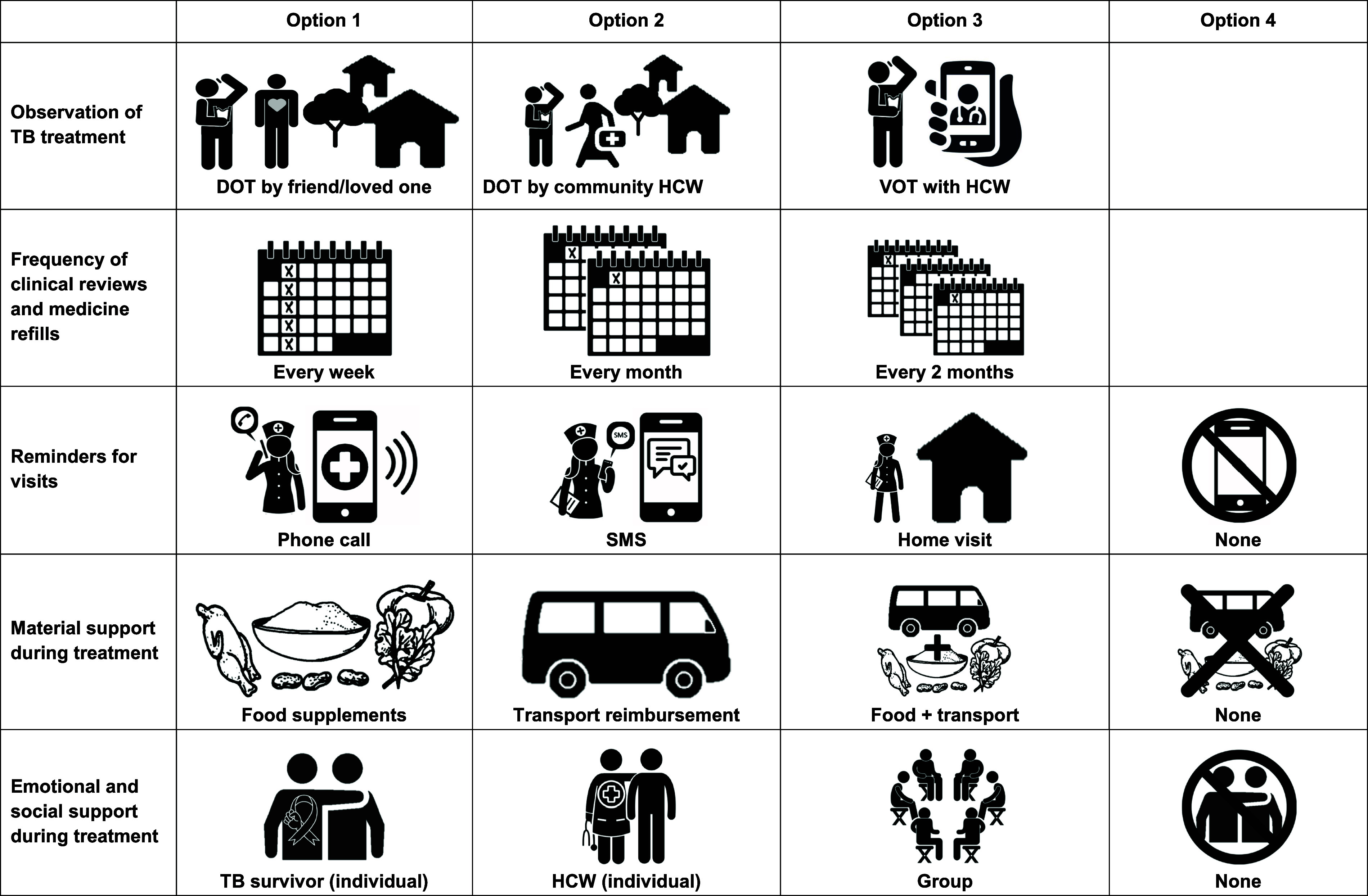
Overview of discrete choice experiment design to elicit preferences for a multidrug-resistant TB treatment support package, showing each feature and their options. DOT = directly observed therapy; VOT = video observed therapy; HCW = health care worker.

For the DCE, the support package features (i.e., attributes) and their options (i.e., levels) were selected through a structured process that included a review of literature on MDR-TB treatment support and relevant WHO guidance, which generated a list of candidate features (and options) that was consolidated through iterative discussions with the study team, MDR-TB providers, and implementing partners to ensure local relevance and feasibility while minimising cognitive burden for participants.^14^ Five features with three to four options each were included in the final DCE design: frequency of visits/refills, type of treatment observation, visit reminders, emotional and social support, and material support (referred to as ‘physical support’ in participant-facing materials for greater clarity – see Figure 1). To enhance comprehension, particularly among participants with limited literacy, the support package feature options were represented using icons (Figure 1), and an educational flipbook with a standardised description of the exercise and the different features (and options) was reviewed before starting the DCE (Supplementary Data S1). The DCE and flipbook instrument was piloted with four representative participants to assess understanding, ease of completion, and cognitive burden. Based on pilot feedback, minor adjustments were made to the translated explanations to improve comprehension. The finalised DCE was programmed in Lighthouse Studio version 14.1.2 (Sawtooth Software) using a near-balanced overlap design.^15^

Eligible participants were enrolled and completed the DCE during their routine treatment follow-up visits. The DCE was facilitated on an electronic tablet by a trained research staff member. After reviewing the flipbook and completing a practice task, participants were presented with 12 randomised choice tasks. Each task required participants to choose between two hypothetical MDR-TB support packages (labelled as Option 1 or 2) based on their assessment of the different feature options presented (Supplementary Data S2 shows an example choice task). To evaluate comprehension and engagement, participants completed one fixed task that showed a clearly superior support package option,^16^ as well as three Likert-style questions after completion to assess perceived difficulty of the exercise.

### Statistical analysis

The sample size was calculated using the formula: 500 I/(JS), where I is the largest number of levels for any one feature, J is the number of alternatives presented in a choice task, and S is the total number of choice tasks. Based on our design, the minimum sample size was 83 participants (500[4]/[2 × 12]).^17^ However, we targeted enrolment of 100 participants, which was approximately equal to the number of persons initiated on MDR-TB treatment in Lusaka District the prior year. Prior to analysis, we applied several measures to ensure high-quality individual-level responses.^17–19^ We excluded participants who had incomplete data, failed the dominant choice task, or always selected either option A or option B in every choice task (i.e., straightlining). We also excluded respondents whose root likelihood (RLH) values fell below the 95% threshold for random responders (0.706, based on 500 simulated random-response datasets)^18^ and who self-reported difficulty understanding the choice tasks or distinguishing between options. Hierarchical Bayesian models were used to estimate zero-centred individual-level and overall mean preference weights (MPWs) of options for different support features.^17^ The relative importance (RI) of support features was calculated as the range of MPWs for a given feature divided by the sum of all ranges across features. The primary analysis focused on the overall MPW and RI. Secondarily, we conducted a latent class analysis to identify distinct preference groups according to heterogeneity in preferences for support package features. The optimal number of groups was determined by maximising statistical fit while considering minimum group size requirements and interpretability (Supplementary Data S3).^18,20,21^ Analyses were conducted using Lighthouse Studio version 14.1.2 and Stata 17.0 (StataCorp LLC).

### Ethical statement

Institutional approval was obtained from the University of Zambia Biomedical Research Ethics Committee (#3903-2023). All participants provided written informed consent in their preferred language (Nyanja, Bemba, or English).

## RESULTS

Of 102 individuals enrolled, 3 did not meet pre-defined data quality standards (1 had incomplete data and 2 failed the dominant choice task); therefore, 99 were included in the final analysis. Participants had a median age of 36.0 years, 68.9% were male, 42.4% were HIV-positive, and 47.5% reported a previous history of TB (Table 1). The majority (57.6%) reported an annual income below 8,000 Kwacha (approximately US$1 per day), and 32.3% lived more than 5 km away from their TB treatment facility. Nearly all participants (95.0%) were receiving a longer-course MDR-TB treatment regimen. No participants indicated that the DCE was difficult to understand or that it was difficult to distinguish between the two hypothetical support package options in each choice task.

**Table 1.** Participant characteristics overall and by latent class preference group.

	Overall (n = 99; 100%)	Group 1 – ‘material support dominant’ (n = 39; 39.4%)	Group 2 – ‘balanced priorities’ (n = 31; 31.3%)	Group 3 – ‘visit frequency sensitive’ (n = 29; 29.3%)	*P* value
Age, median (IQR)	36.0 (29.2–43.1)	36.0 (27.8–43.9)	38.2 (31.0–42.5)	32.0 (27.1–41.4)	0.58
Age group					
<30	26 (26.3)	12 (30.8)	6 (19.4)	8 (27.6)	0.77
30–49.9	62 (62.6)	24 (61.5)	21 (67.7)	17 (58.6)	
50+	11 (11.1)	3 (7.7)	4 (12.9)	4 (13.8)	
Sex					
Male	68 (68.9)	23 (59.0)	24 (77.4)	21 (72.4)	0.24
Female	31 (31.3)	16 (41.0)	7 (22.6)	8 (27.6)	
Marital status					
Single	33 (33.3)	13 (33.3)	10 (32.3)	10 (34.5)	0.64
Married	33 (33.3)	13 (33.3)	13 (41.9)	7 (24.1)	
Divorced/separated/widowed	33 (33.3)	13 (33.3)	8 (25.8)	12 (41.4)	
Highest education level					
None	9 (9.1)	4 (10.3)	5 (16.1)	0	0.21
Primary	25 (25.3)	13 (33.3)	6 (19.4)	6 (20.7)	
Secondary	58 (58.6)	20 (51.3)	17 (54.8)	21 (72.4)	
University/college	7 (7.1)	2 (5.1)	3 (9.7)	2 (6.9)	
Income per year in Zambian kwacha (ZMW)					
<4,000 (<$0.5/day)	26 (26.3)	10 (25.6)	13 (41.9)	3 (10.3)	0.025
4,000–8,000 ($0.5–$1.0/day)	31 (31.3)	13 (33.3)	4 (12.9)	14 (48.3)	
8,000–20,000 (∼$1.0 to $2.3/day)	33 (33.3)	12 (30.8)	10 (32.3)	11 (37.9)	
>20,000 (∼$2.3/day)	9 (9.1)	4 (10.3)	4 (12.9)	1 (3.5)	
HIV status					
Positive	42 (42.4)	16 (41.0)	17 (54.8)	9 (31.0)	0.19
Negative	57 (57.6)	23 (59.0)	14 (45.2)	20 (69.0)	
Prior TB disease					
Yes	47 (47.5)	14 (35.9)	16 (51.6)	17 (58.6)	0.15
No	52 (52.5)	25 (64.1)	15 (48.4)	12 (41.4)	
Distance to health facility from home in kilometres					
≤5	67 (67.7)	25 (64.1)	25 (80.7)	17 (58.6)	0.20
6–10	19 (19.2)	7 (18.0)	3 (9.7)	9 (31.0)	
>10	13 (13.1)	7 (18.0)	3 (9.7)	3 (10.3)	
Treatment regimen type					
Long-course	94 (95.0)	37 (94.5)	29 (93.6)	28 (96.6)	1
Short-course	5 (5.1)	2 (6.1)	2 (6.5)	1 (3.5)	
Time since treatment initiation in months, group					
<1	22 (22.2)	6 (15.4)	9 (29.0)	7 (24.1)	0.47
1–2.9	24 (24.2)	7 (18.0)	10 (32.3)	7 (24.1)	
3–5.9	11 (11.1)	6 (15.4)	2 (6.5)	3 (10.3)	
6–11.9	42 (42.4)	20 (51.3)	10 (32.3)	12 (41.1)	

Material support was the most important feature of treatment support packages (RI = 45.7%, 95% confidence interval [CI]: 43.6–47.8), being 1.7 to 9.7 times more important than any other feature. The frequency of clinic visits and refills ranked as the second most valued feature (RI = 26.7%, 95% CI: 25.1–28.3), followed by the mode of visit reminders (RI = 11.8%, 95% CI: 10.5–13.1), the mode of emotional and social support (RI = 11.2%, 95% CI: 10.0–12.4), and the mode of treatment observation (RI = 4.7%, 95% CI: 4.0–5.4). For material support, the combined provision of food and transport reimbursement was the most preferred option (MPW = 110.1), followed by transport reimbursement alone (MPW = 18.5) and food supplements alone (MPW = −10.3) compared to no material support (MPW = −118.4). For the less valued features, monthly (MPW = 39.9) and bimonthly (MPW = 42.4) visits were similarly preferred, phone calls were the most preferred form of visit reminders (MPW = 21.5), health care worker (HCW)-based support was the most preferred form of emotional and social support (MPW = 25.2), and DOT in the community with a community HCW (MPW = 3.8) or a friend/loved one (MPW = 3.2) was similarly preferred.

**Figure 2.**
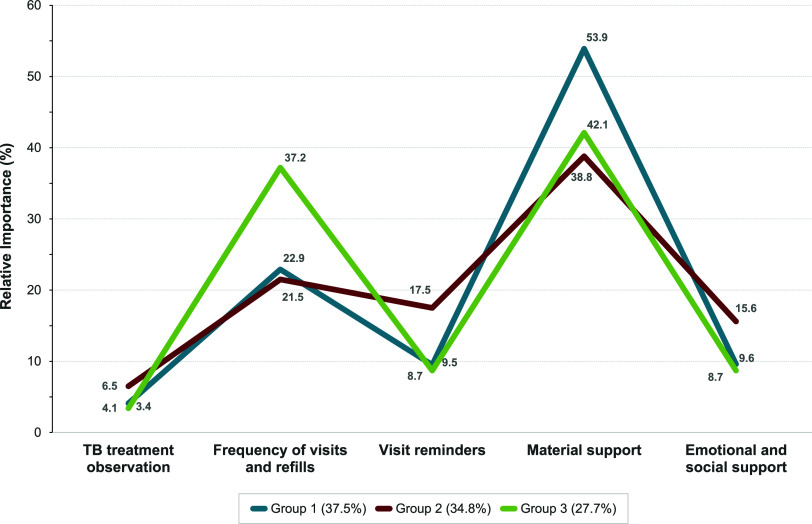
The relative importance of features for a TB treatment support package among people with multidrug-resistant TB according to latent class preference group. Values represent the percentage importance attributed to each feature (summing to 100% within each group), with higher values indicating greater influence on preferences. Each coloured line represents a distinct preference group.

Latent class analysis identified three distinct preference groups (Figure 2, Table 2). All groups most highly prioritised material support, though the importance of other features and their delivery varied across classes. Group 1 (n = 39; 39.4%) placed the greatest importance on material support across the three groups (‘Material support dominant’), Group 2 (n = 31; 31.3%) placed more balanced importance on visit frequency, visit reminders, and emotional/social support (‘Balanced priorities’), while Group 3 (n = 29; 29.3%) placed greater importance on frequency of visits/refills (‘Visit frequency sensitive’). Across groups, modality-level preferences were relatively consistent: participants preferred community-based DOT delivered by either a community HCW or a trusted loved one; phone call- or SMS-based reminders; and, when only one form of material support could be offered, transport reimbursement over food supplements (particularly in Groups 1 and 3). Emotional support from an HCW was most favoured across all groups, with additional small positive preferences for group support in Group 1 and TB survivor support in Group 2. Notably, all groups showed strong negative preferences for video observed therapy (VOT), weekly visits, visit reminders via home-based outreach or no reminders, as well as no material or social support. The demographic profiles of the three preference groups were largely similar except for income (Table 1), with Group 2 having a higher proportion of participants in the lowest income category (<4,000 Kwacha per year).

**Table 2.** Mean preference weights for different feature options of a multidrug-resistant TB treatment support package, overall and according to latent class preference group.

	Overall (n = 99)	Group 1 – ‘material support dominant’ (n = 39; 39.4%)	Group 2 – ‘balanced priorities’ (n = 31; 31.3%)	Group 3 – ‘visit frequency sensitive’ (n = 29; 29.3%)
TB treatment observation				
DOT – community HCW	3.8 (1.5–6.2)	4.8 (1.8–7.8)	3.3 (−2.9 to 9.5)	3.1 (0.8–5.5)
DOT – loved one	3.2 (1.4–5.0)	3.4 (1.6–5.2)	3.1 (1.6–5.2)	3.1 (0.3–5.8)
VOT	−7.1 (−9.4 to −4.7)	−8.2 (−11.1 to 5.4)	−6.4 (−12.6 to −0.1)	−6.2 (−8.9 to 3.4)
Frequency of visits and refills				
Every week	−82.2 (−89.5 to −75.0)	−72.8 (−79.0 to −66.7)	−58.4 (−72.4 to −44.3)	−120.1 (−125.3 to −115.3)
Every month	39.9 (36.2–43.6)	36.2 (32.9–39.6)	28.7 (20.8–36.7)	56.6 (53.4–59.9)
Every 2 months	42.4 (37.8–46.9)	36.6 (32.5–40.8)	29.6 (19.7–39.6)	63.7 (60.4–66.9)
Visit reminders				
Phone reminder	21.5 (18.3–24.8)	15.8 (13.1–18.4)	35.6 (28.5–42.8)	14.3 (10.7–17.9)
SMS reminder	15.0 (11.9–18.0)	12.3 (9.2–15.4)	20.4 (12.3–28.5)	12.8 (9.3–16.2)
Home visit reminder	−8.0 (−13.4 to −4.5)	−2.1 (−6.5 to 2.2)	−22.5 (−33.9 to −11.1)	−3.6 (−7.3 to 0.2)
No reminder	−27.6 (−30.1 to −25.0)	−25.9 (−28.5 to −23.3)	−33.5 (−40.3 to −26.7)	−23.5 (−25.7 to −21.3)
Material support				
Food supplements	−10.3 (−13.8 to −6.8)	−16.6 (−20.1 to −13.1)	5.4 (−2.1 to 13.0)	−18.6 (−21.4 to −15.8)
Transport reimbursement	18.5 (15.3–21.9)	30.6 (27.3–33.9)	4.3 (−1.9 to 10.5)	17.7 (15.4–33.9)
Food + transport	110.1 (105.3–114.9)	127.6 (122.7–132.6)	92.2 (82.6–101.9)	105.6 (101.2–110.0)
No material support	−118.4 (−124.2 to −112.7)	−141.7 (−146.5 to −136.8)	−102.0 (−113.6 to −90.3)	−104.7 (−109.7 to −99.7)
Emotional and social support				
TB survivor support	2.6 (−0.6 to 5.7)	−5.4 (−7.7 to −3.0)	15.9 (8.6–23.2)	−1.1 (−4.1 to 1.9)
HCW support	25.2 (23.0–27.3)	20.7 (18.4–23.1)	31.8 (26.7–37.0)	24.1 (22.1–26.0)
Group support	−3.0 (−6.6 to 0.7)	9.9 (6.6–13.2)	−15.8 (−23.8 to −7.8)	−6.5 (−9.2 to 3.8)
No social support	−24.8 (−28.5 to −21.1)	−25.2 (−23.0 to −21.5)	−31.9 (−41.4 to −22.4)	−16.5 (−20.5 to −12.4)

DOT = directly observed therapy; VOT = video observed therapy; HCW = health care worker.

## DISCUSSION

This study provides robust evidence about which components of MDR-TB treatment support packages are most valued by urban-dwelling individuals receiving care in Lusaka, Zambia. Overall, and across preference groups, participants consistently prioritised material support – particularly the combined provision of transport reimbursement and food supplementation – and expressed strong preferences for reduced clinic visit frequency. These findings underscore how MDR-TB care intersects with individuals’ daily survival needs and reinforce the central role that person-centred support must play in improving treatment engagement in high-burden settings.

The overwhelming importance placed on material support reflects the substantial economic challenges faced by people with MDR-TB.^4,22^ Although the lowest income participants were concentrated in Group 2, who showed more balanced preferences for several treatment support features, nearly 60% of participants overall were living below 8,000 Kwacha annually (∼1 USD/day), reflecting widespread economic vulnerability and likely explaining why material support predominated across all groups. Lengthy treatment durations often lead to income loss or employment difficulties, while medication requirements and side effects can increase nutritional needs. The very strong disutility for no material support across all latent classes highlights a foundational requirement: without direct material support, even well-designed clinical or adherence interventions may have limited impact. These findings align with the WHO’s call for universal patient support and social protection in TB care.^23,24^ Notably, when only one form of material support could be offered, transport reimbursement was preferred over food supplements despite most participants living within 5 km of their treatment facility. A DCE in Uganda similarly found travel vouchers to be the most valued form of support, with participants noting that remaining funds could be used for food, household necessities, and debt repayment.^25^ Together, these findings suggest that cash-based reimbursement may offer perceived flexibility beyond its stated purpose, while also addressing the immediate, recurring nature of transport costs with each clinic visit.

Preferences for less frequent clinic visits further reflect the considerable burden that facility attendance places on patients. In the early phases of treatment, many individuals are physically debilitated from MDR-TB disease itself, making frequent travel to clinics exhausting and difficult. As treatment progresses and individuals recover, competing demands emerge – even if transport costs are reimbursed, frequent clinic visits can disrupt employment, family responsibilities, and daily routines. The absence of meaningful difference in the preferences of people with MDR-TB between monthly and bimonthly visits represents an actionable finding: programmes have genuine implementation flexibility, and either interval appears acceptable as long as weekly visits are avoided. This supports differentiated service delivery models that reduce visit frequency while maintaining adequate clinical monitoring.^26–28^

Participants showed clear preferences regarding how additional types of treatment support are delivered.^2^ Notably, participants valued some form of emotional or social support; while HCWs were the most preferred source of emotional support across groups, some preference groups showed additional positive preferences for group-based support or TB survivor-led support. The overall preference for HCW-led emotional support likely reflects established trust and greater perceived knowledge and confidentiality, or limited familiarity and/or comfort with TB survivor and group support models among perceived peers in this setting. For visit reminders, phone calls were slightly preferred over SMS, likely reflecting the immediacy and personalisation of voice communication, but both appeared acceptable. For directly observed treatment, community-based models delivered by either a community health worker or a trusted friend or family member were similarly acceptable, suggesting that convenient, supportive, and non-intrusive observation models may matter more than the specific provider type. Equally notable, participants showed strong disutility for home-based outreach reminders and VOT, suggesting these are unacceptable options in this setting. Negative preferences for home visits likely reflect concerns about privacy and TB-related stigma, while negative preferences for VOT may reflect practical barriers such as limited smartphone or data access, distrust of a new technology, or discomfort with video-based monitoring.

These preference insights have important implications for MDR-TB programmes in Zambia and similar high-burden settings and should inform how support packages are designed and delivered. Support packages should prioritise financial protection as a core therapeutic intervention, not an auxiliary component.^24^ Programmes should provide integrated food and transport support, rather than offering these services selectively or intermittently. They should adopt longer refill intervals wherever clinically appropriate and programmatically feasible, aligning care delivery with the preferences of people with MDR-TB and minimising opportunity costs. Additional flexible support options – whether for DOT supporter (community health worker or trusted loved one), visit reminder modality (phone call or SMS), or psychosocial support (HCW-led, group-based, or TB survivor-led) – should be provided when feasible to accommodate the diverse preferences observed in latent class analyses, while clearly unacceptable modalities such as VOT and home-based outreach should be avoided.

This study has several strengths, including enrolment across six treatment facilities, the use of pilot testing as well as visuals and an educational flipbook to ensure standardised understanding of the DCE and differing features and their options across participants with varying literacy levels, and latent class analysis to identify distinct preference groups that would have been obscured in aggregate estimates. Limitations include an exclusively urban sample; therefore, preferences in rural or hard-to-reach settings, where structural barriers are potentially more significant, may differ. The modest sample size may have limited the power to detect additional latent classes or affected latent class stability; however, the analysis still identified three groups with distinct preference patterns, providing actionable insights. Additionally, while we worked with local MDR-TB providers to ensure relevance, due to time and resource constraints, we were unable to conduct formative qualitative research with people with MDR-TB to inform the selection of features and their options. Finally, nearly all participants were receiving longer-course MDR-TB regimens (18–24 months); although a shorter regimen was available (4–6 months Bdq, Lfx/Mfx, Lzd, E, Z, Hh, Cfz/5 months Lfx[Mfx]-Cfz-Z-E), it was not routinely initiated due to its higher daily pill burden. Newer shorter regimens with lower daily pill burdens (e.g., 6 months of BPaLM) are increasingly available. The structural drivers of the observed preferences – extreme poverty, transport costs, and competing life demands – are characteristics of TB-affected populations and will not disappear simply because regimens are shorter or require fewer pills. Thus, while we expect these findings to be largely generalisable to shorter regimens (both current and future), whether the RI of different support features differs warrants further study.

In conclusion, people with MDR-TB in Lusaka strongly valued comprehensive material support and reduced clinic visit burden. The consistency of these preferences across preference groups – despite variation in other features – suggests that material support incorporating transport assistance and food supplementation should be a core component of MDR-TB care. These findings can inform the design of future MDR-TB treatment support packages for research and programmatic settings in Zambia and similar high-burden settings.

## Supplementary Material
