# Supplementary material for: Defining person-centred treatment support for multidrug-resistant TB: a discrete choice experiment
Source: IJTLD Open. 2026 Jun 15;3(6):376–83. doi: 10.5588/ijtldopen.25.0825 (PMC13268096; doi:10.5588/ijtldopen.25.0825)
Supplement: Supplementary file 1 [file ijtldopen25-0825_supplementarydata1.pdf]

MDR-TB Patient Preference Study

Participant education flipbook

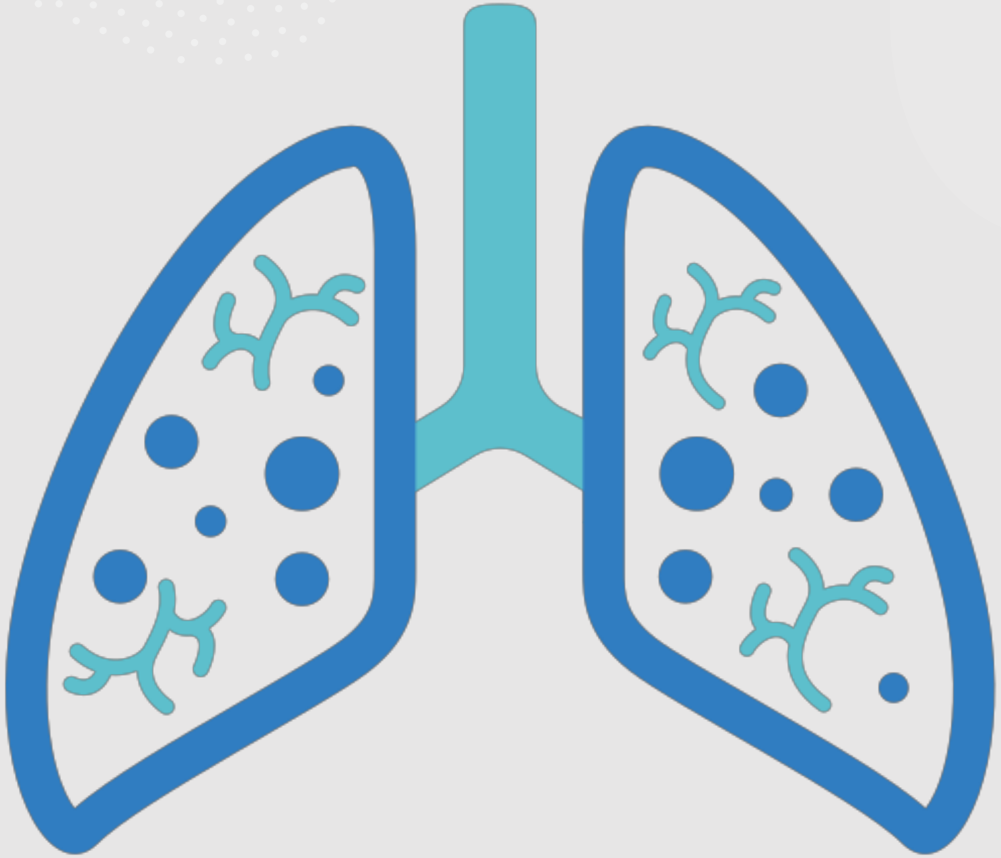

- We want to know what would help you the most while you're taking TB medications for 6 months or more and what forms of treatment support you would most prefer.
- For this survey we've created imaginary ‘TB treatment support packages' to offer different kinds of help and support during the many months of TB treatment. Each package has **5 different types of support**.
- **You'll see 13 questions in total.** For each question we will show you two different 'treatment support packages.' Your job is to pick the one you like the most and that would be the most helpful to you.
- There are no wrong answers as these questions are about what forms of TB treatment support you prefer the most.
- The information from the study will help us develop a package of treatment support that are aligned with what people want and need.

- Please remember, these 'support packages' are imaginary and some types of help or support may not yet exist in Zambia. It also means that your responses will not change your current TB treatment in any way.

- Don’t worry, I’ll now tell you more about the different types of help and support and show you how they survey works.

Question overview

The first column shows the five different types of features that make up the TB treatment support package.

The second and third columns show two different TB treatment support packages. **Your job is to select the one you like the most and would be most helpful to you.**

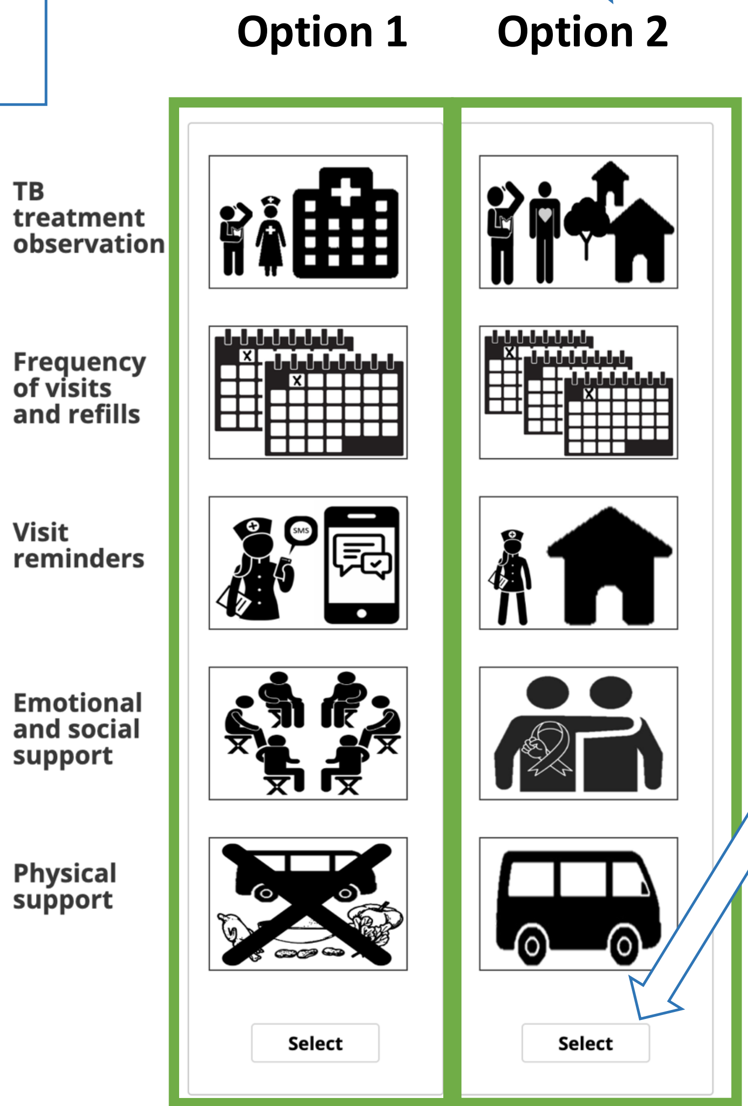

You will see each type of feature appear more than one time in the survey. This is normal. You will still need to consider between the two available options in each question because the combinations will always be different.

TB treatment support features

I will now explain some of the different features of the TB treatment support package to help you better understand the survey.

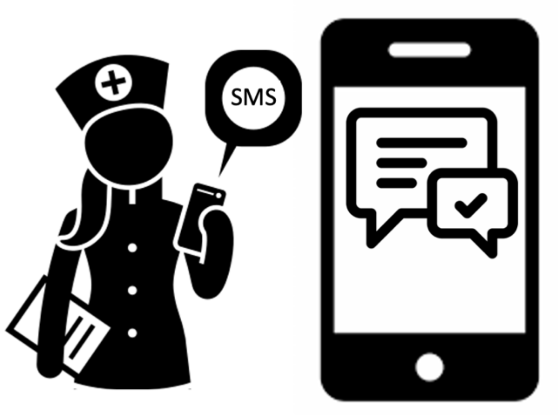

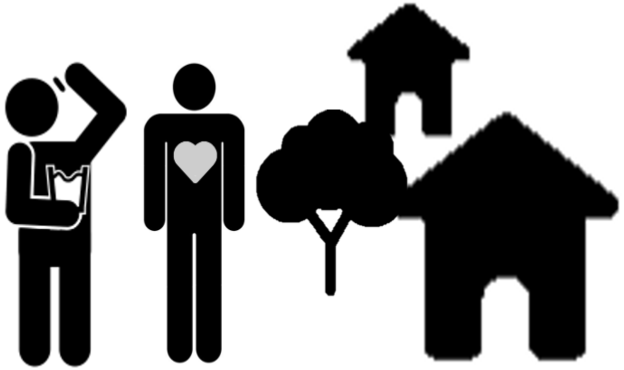

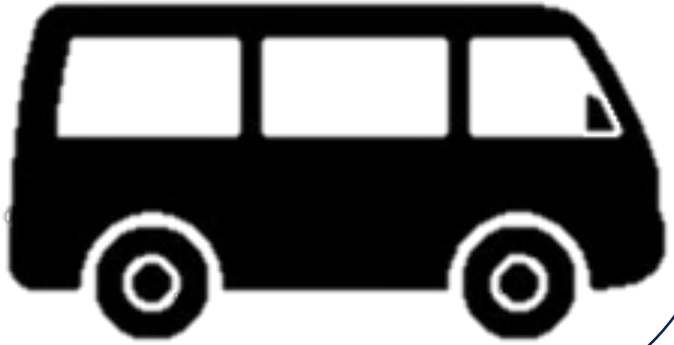

Observation of TB treatment

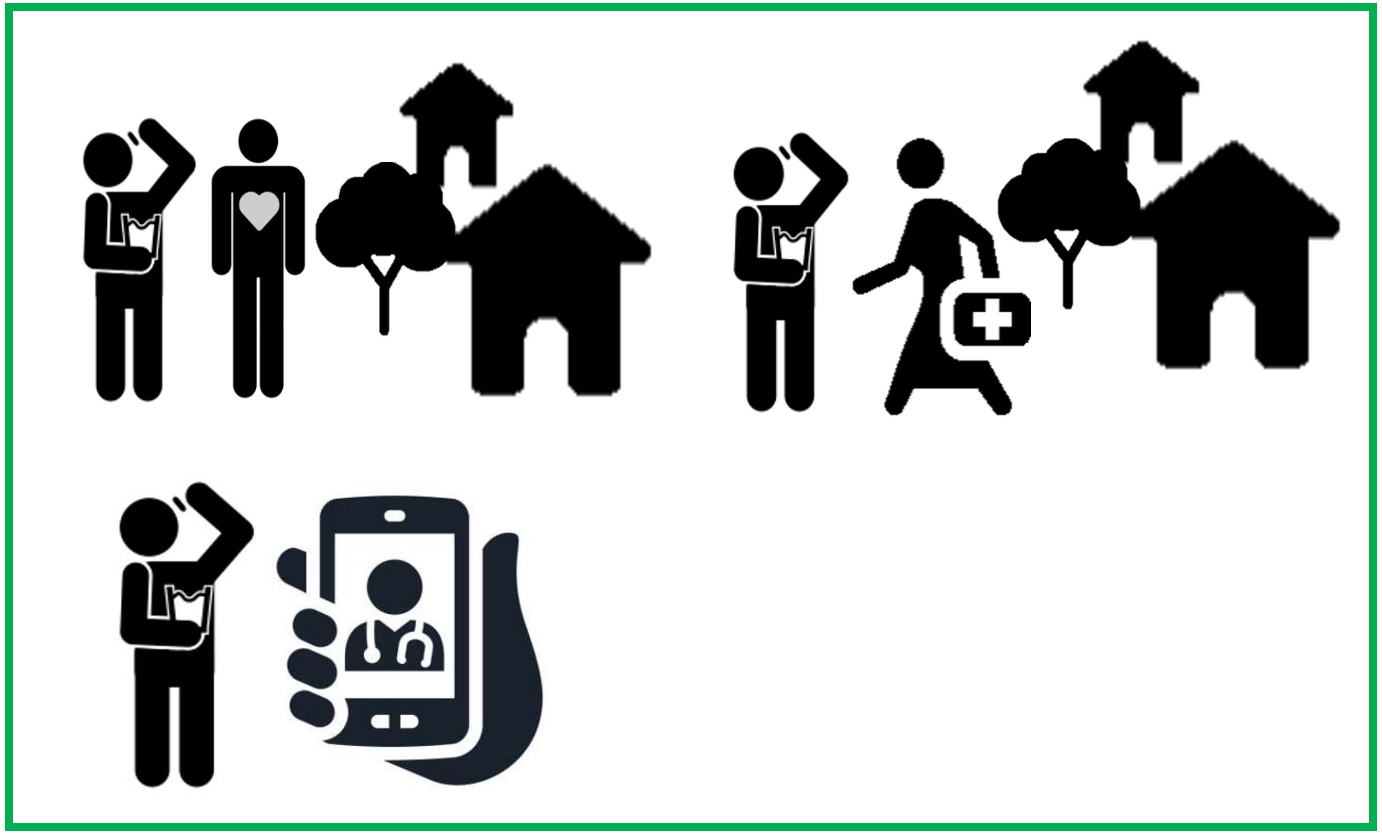

- Usually, a health worker watches a person take their TB medicine every day to make sure they are taking it correctly and consistently.
- We will show you **3 different options** for how you can have your daily medicine observed during TB treatment.

DOT in the community by a friend or loved one

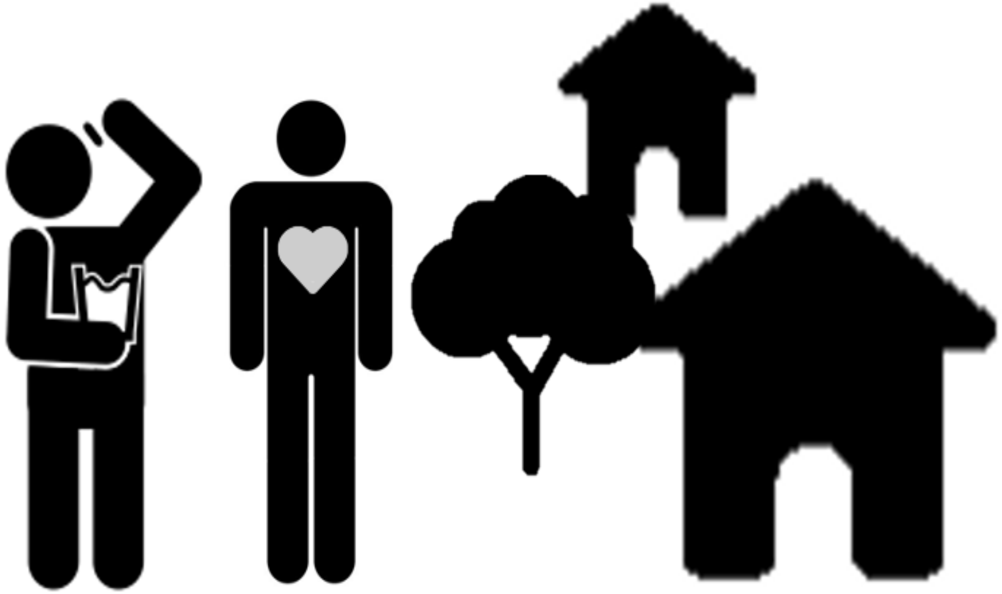

This option means that a friend or loved one will meet you somewhere in the community to watch you take your TB medicine each day. This could be some place close to home or work.

DOT in the community by a community health worker

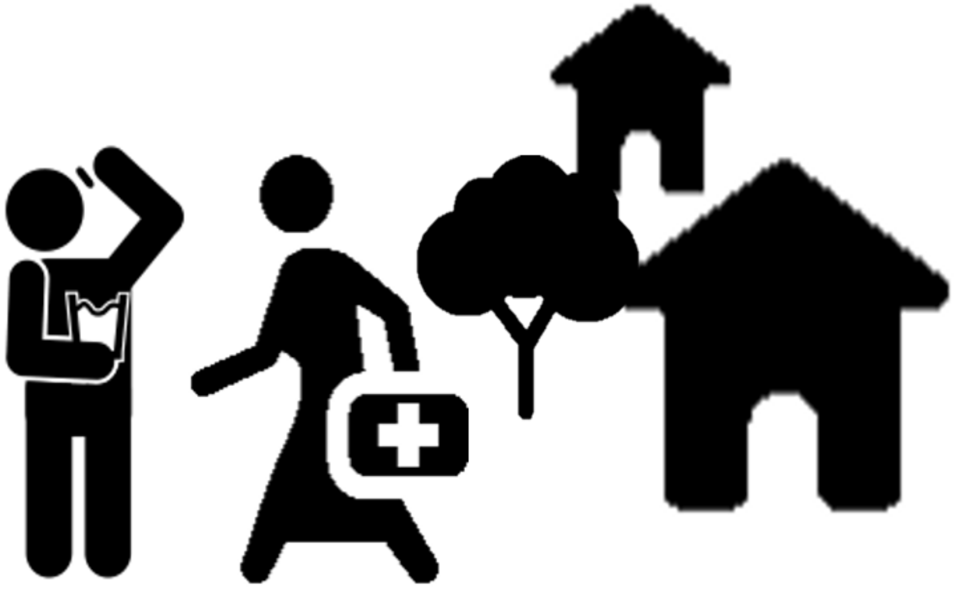

This option means that a community health worker will meet you somewhere in the community to watch you take your TB medicine each day. This could be some place close to home or work.

VOT anywhere with a health worker

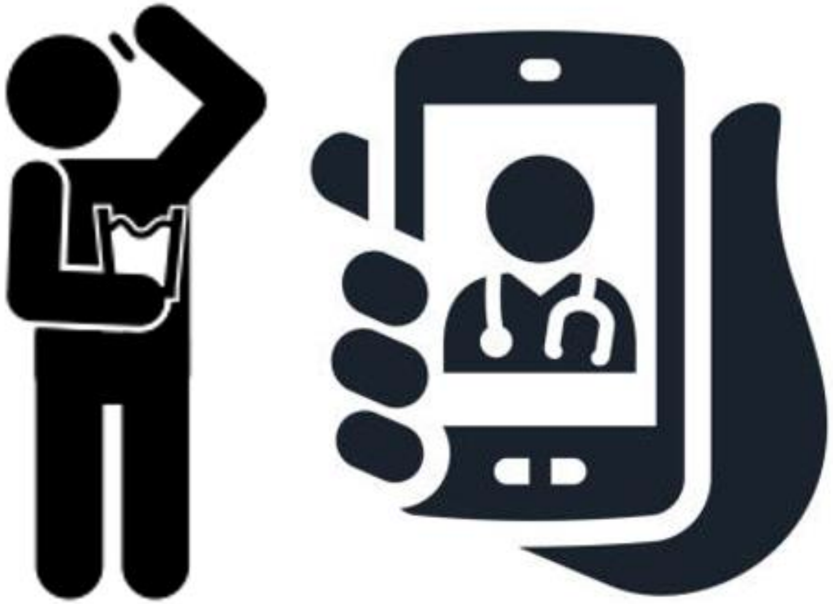

This option means that you can have a video phone call visit with a health worker to watch you take your TB medicine each day. This location could be at home or work, or anywhere else you want.

Frequency of clinical reviews and TB medicine refills

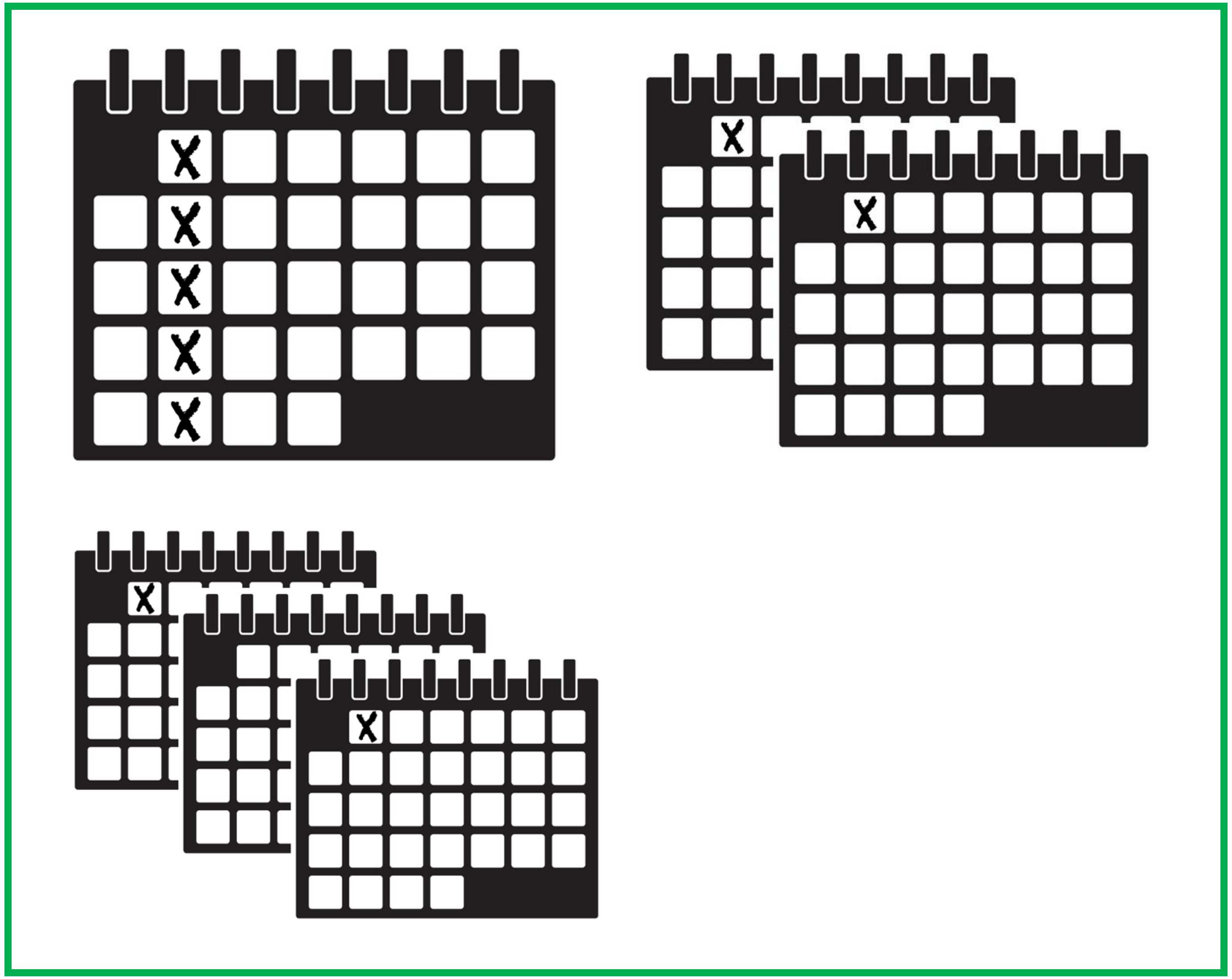

- During TB treatment you come to the health facility to discuss how treatment is going with your team and pick up your TB medicine refills.
- If you are doing daily DOT at the facility, this is a longer visit, and you do not get refills.
- We will show you **3 different options** for how frequently you would need to come to the health facility for this process during TB treatment.

Visits and refills every week

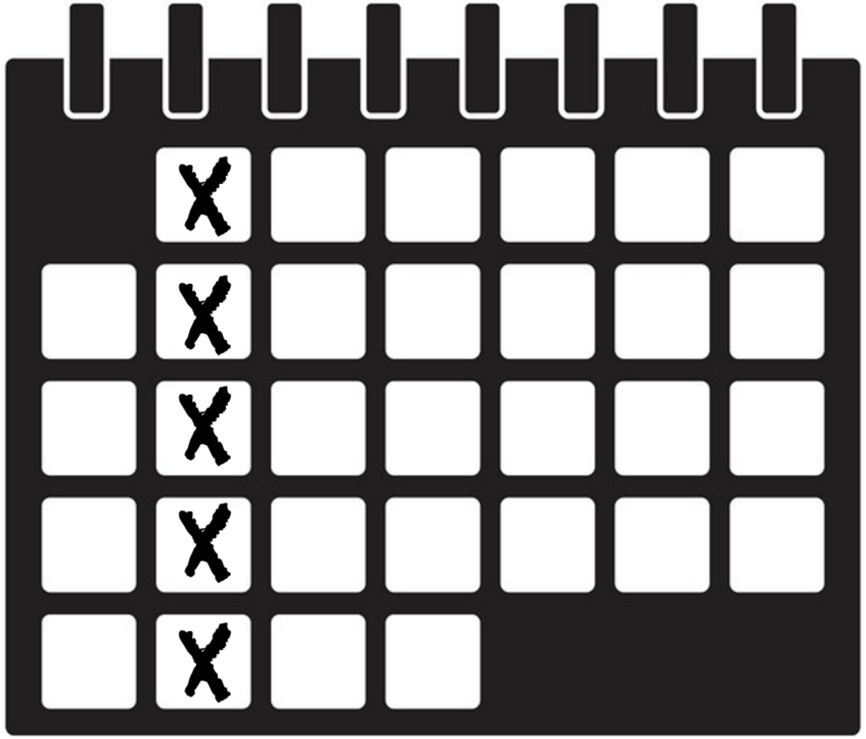

This option means that you must come to the health facility once a week for an appointment to review how your TB treatment is going. You will also pick up your TB medicine meaning, that you get one week of medicine at a time.

Visits and refills every month

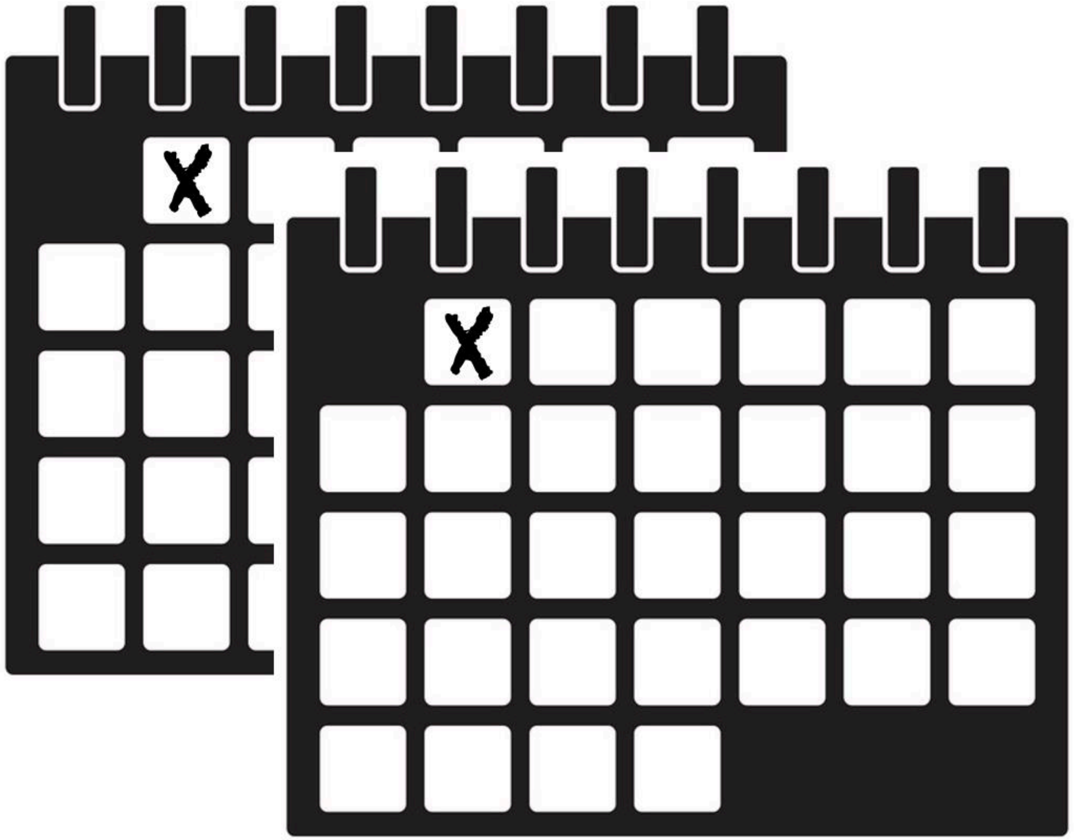

This option means that you must come to the health facility once a month for an appointment to review how your TB treatment is going. You will also pick up your TB medicine meaning, that you get one month of medicine at a time.

Visits and refills every 2 months

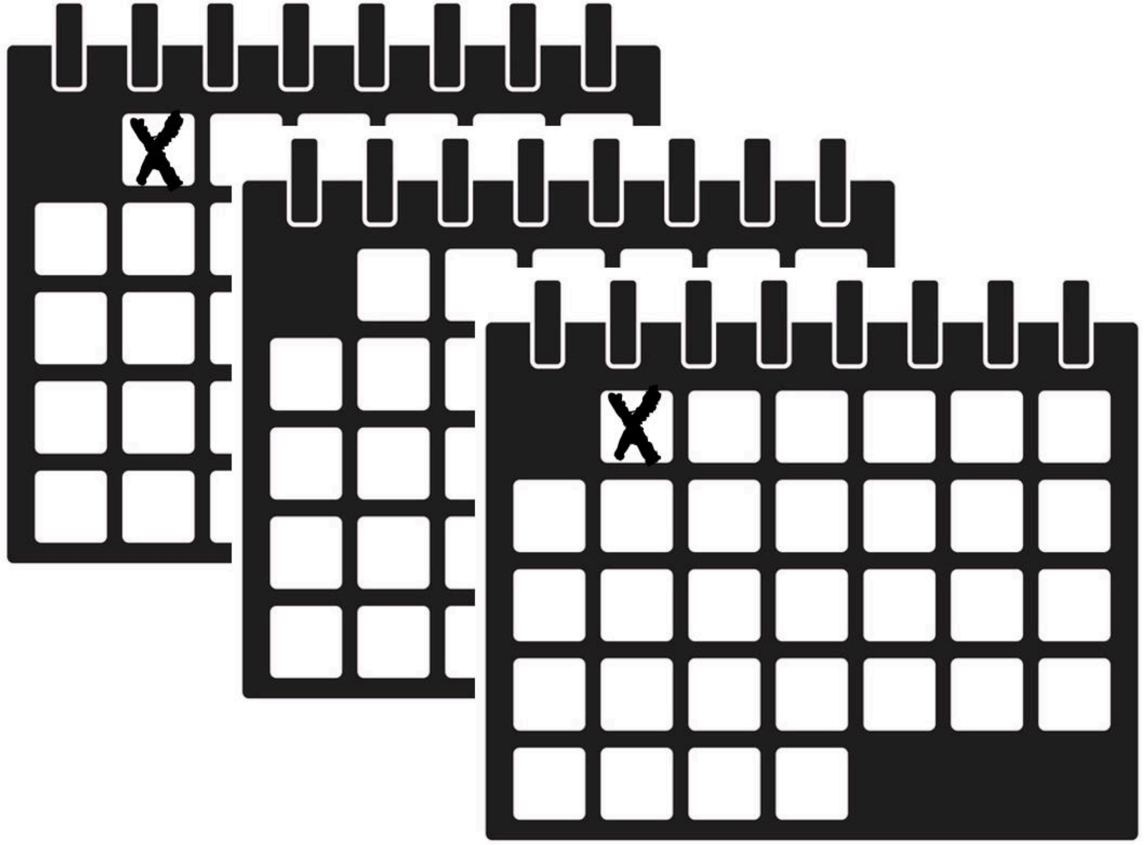

This option means that you must come to the health facility once every 2 months for an appointment to review how your TB treatment is going. You will also pick up your TB medicine meaning, that you get 2 months of medicine at a time.

Reminders for health facility

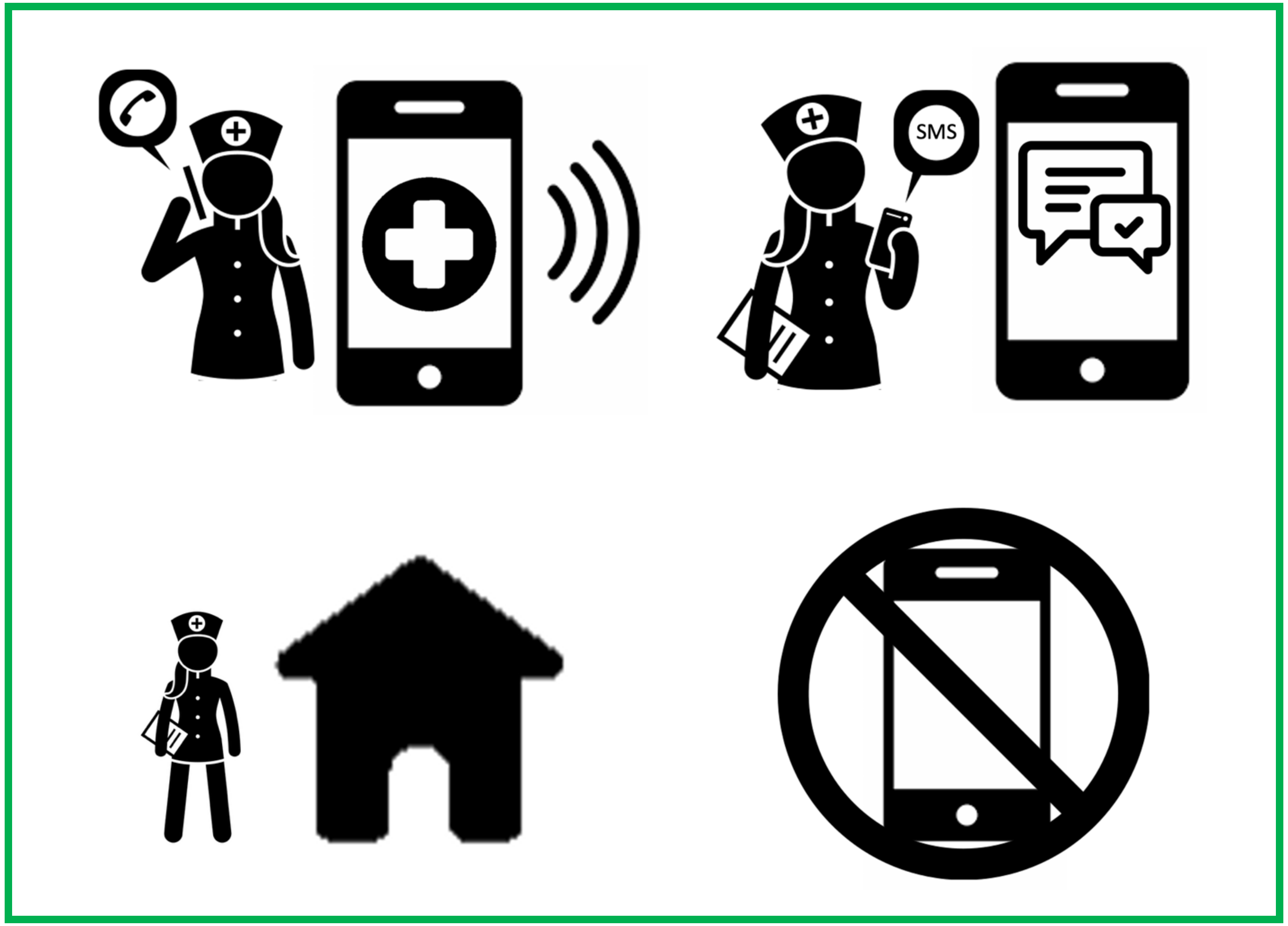

- Before each visit to the health facility to discuss your TB treatment and pick up your medicine refills, you can receive a reminder, so that you do not miss your appointment.

- We will show you **4 different options** for reminders about upcoming appointments at the health facility during TB treatment.

Phone call before appointment

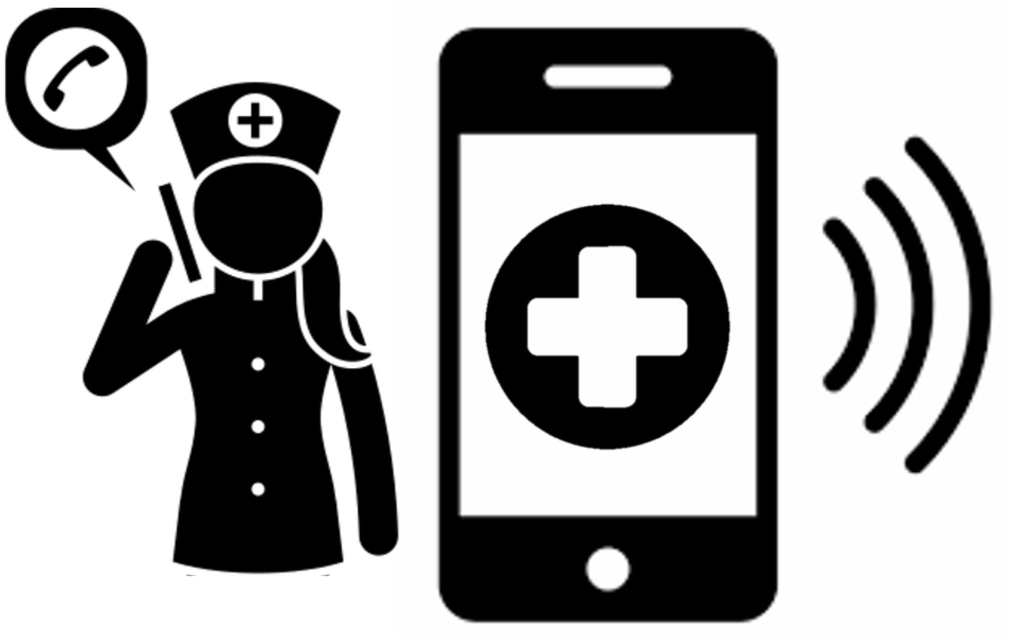

This option means that you will receive a phone call from the clinical team at the health facility before each appointment to help you remember.

SMS before appointment

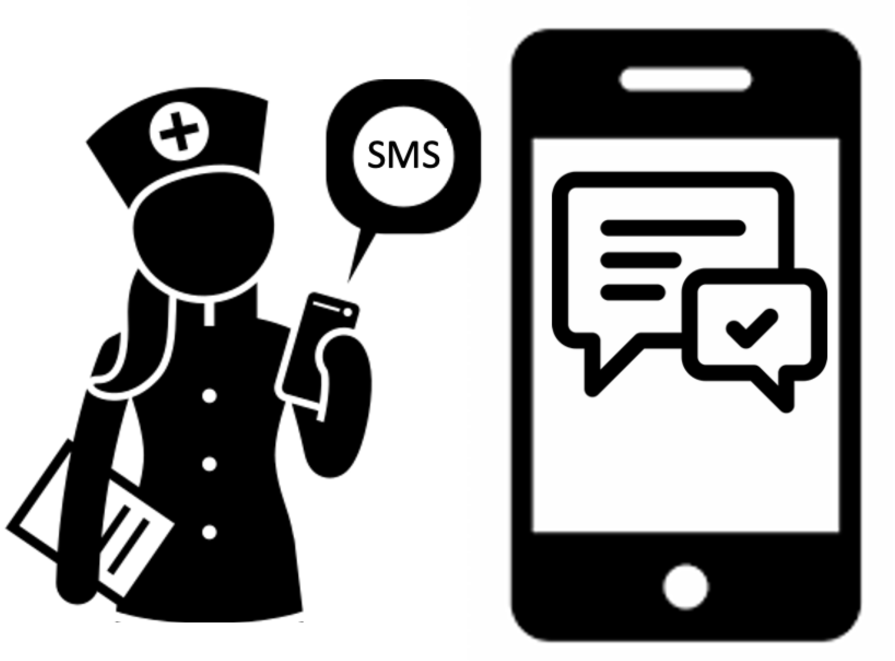

This option means that you will receive a phone call from the clinical team at the health facility before each appointment to help you remember.

Home visit reminder

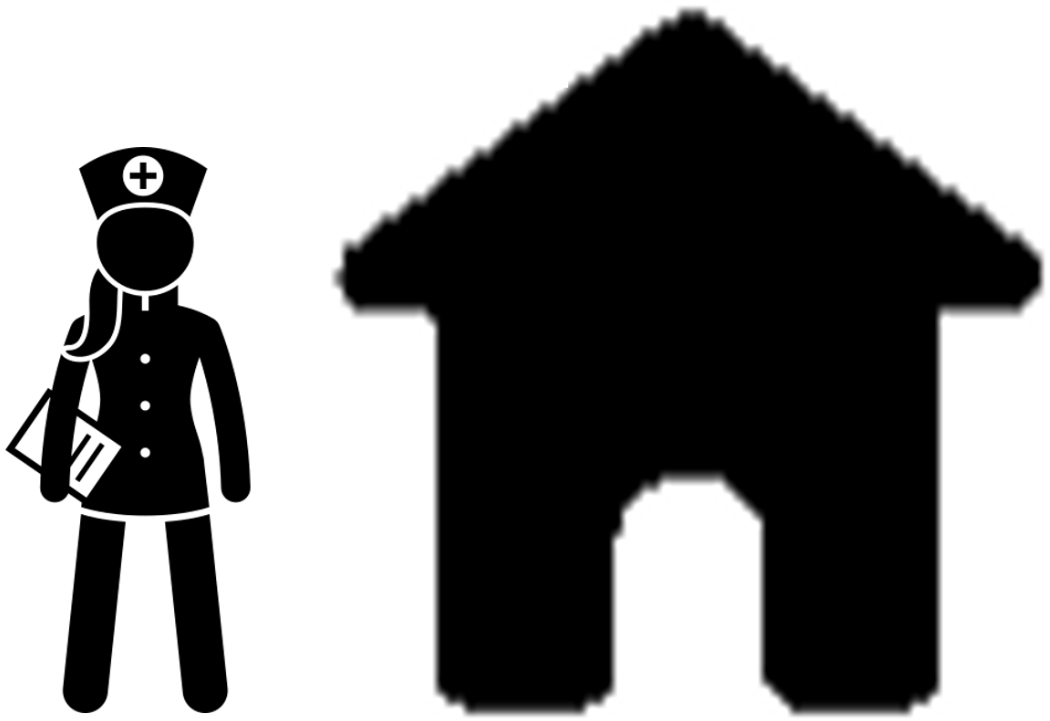

This option means that a community health worker will visit you at home before each appointment to help you remember.

No reminder before appointment

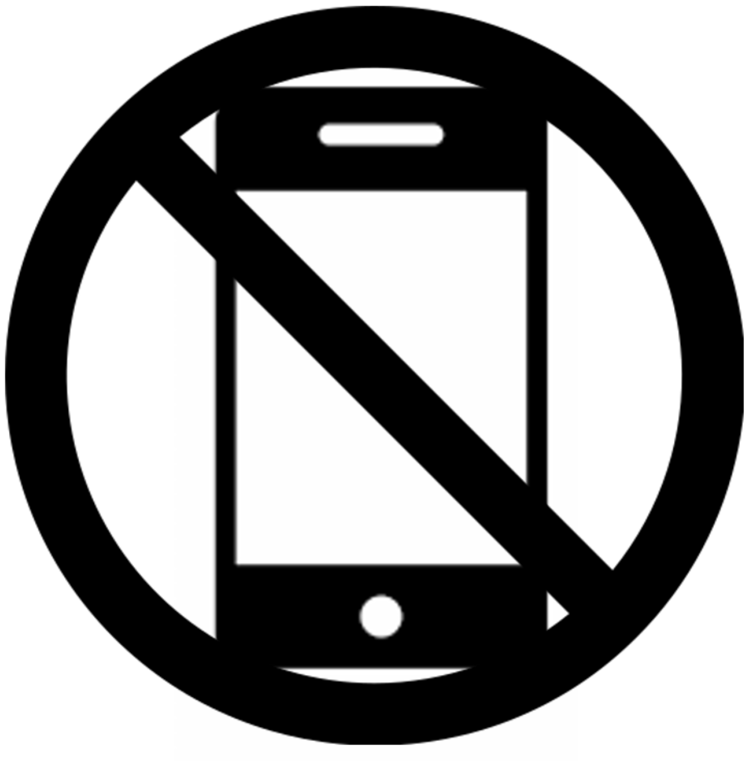

This option means that you will not receive any type of reminder before your appointment at the health facility. You will need to remember the date and time yourself.

Emotional and social support during TB treatment

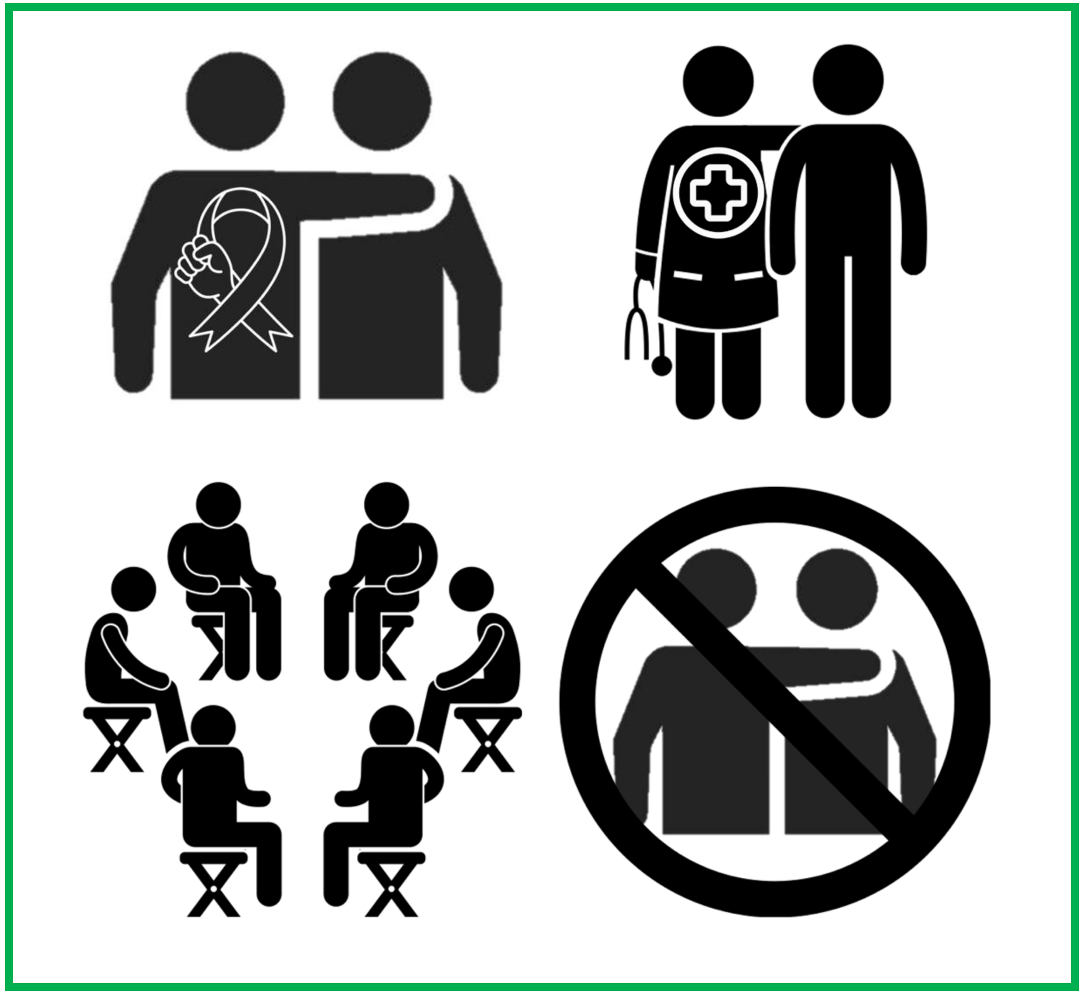

- Getting treated for TB can be very hard sometimes. Having emotional and social support means someone is there to help you deal with challenges, make sure you never feel alone, and will help you stay positive and keep up with your medicine every day.
- We will show you **4 different options** for emotional and social support you can receive during TB treatment.

Individual support from a TB survivor

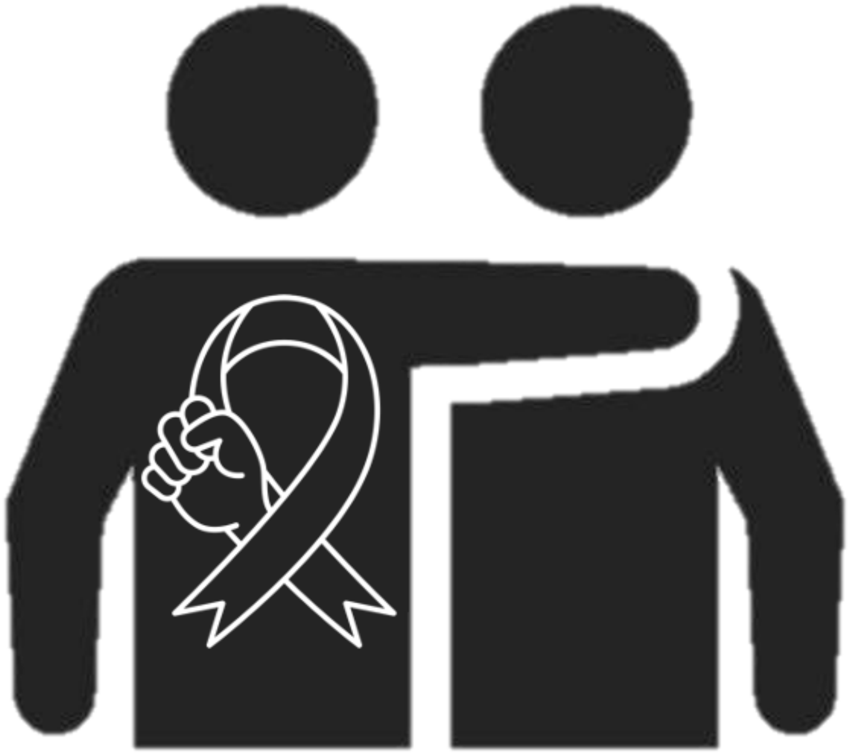

- This option means you can have support from someone who has experienced what you are going through because they had TB and got better.
- This person can be there for you, offer emotional and social support, help you cope with feelings and situations that arise during your TB treatment, and answer your questions.

Individual support from a health worker

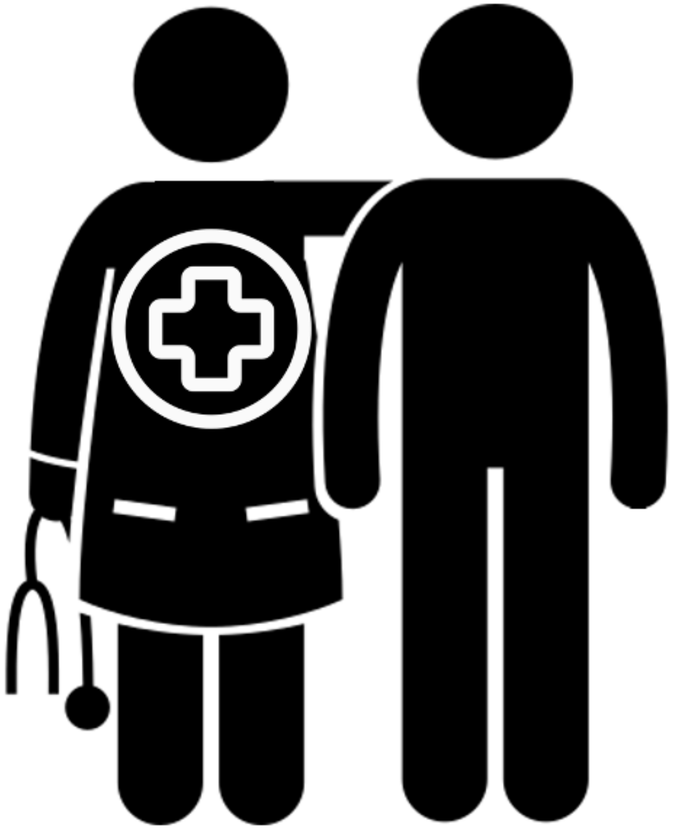

- This option means you can have support from an experienced health worker who has helped others who have experienced what you are going through.
- They have not had TB before.
- This person can be there for you, offer emotional and social support, help you cope with feelings and situations that arise during your TB treatment, and answer your questions.

Group support

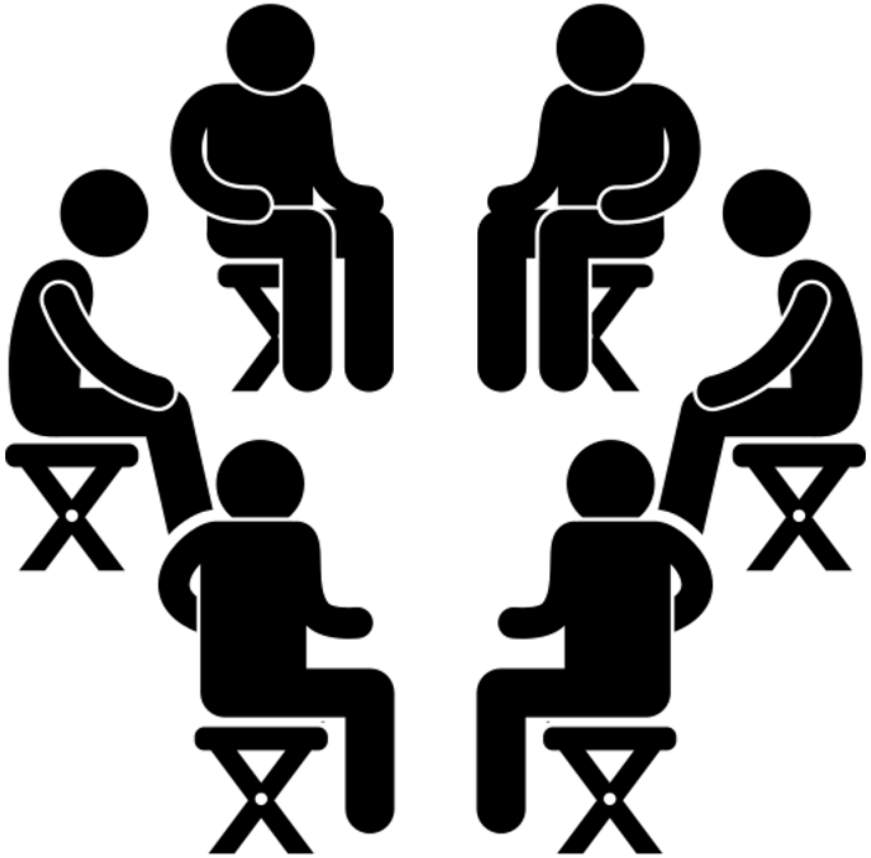

- This option means you meet regularly with a group of individuals who are either being treated for TB or have had TB in the past and therefore know what you are going through.
- This group can be there for you, offer emotional and social support, help you cope with feelings and situations that arise during your TB treatment, and answer your questions.

No support

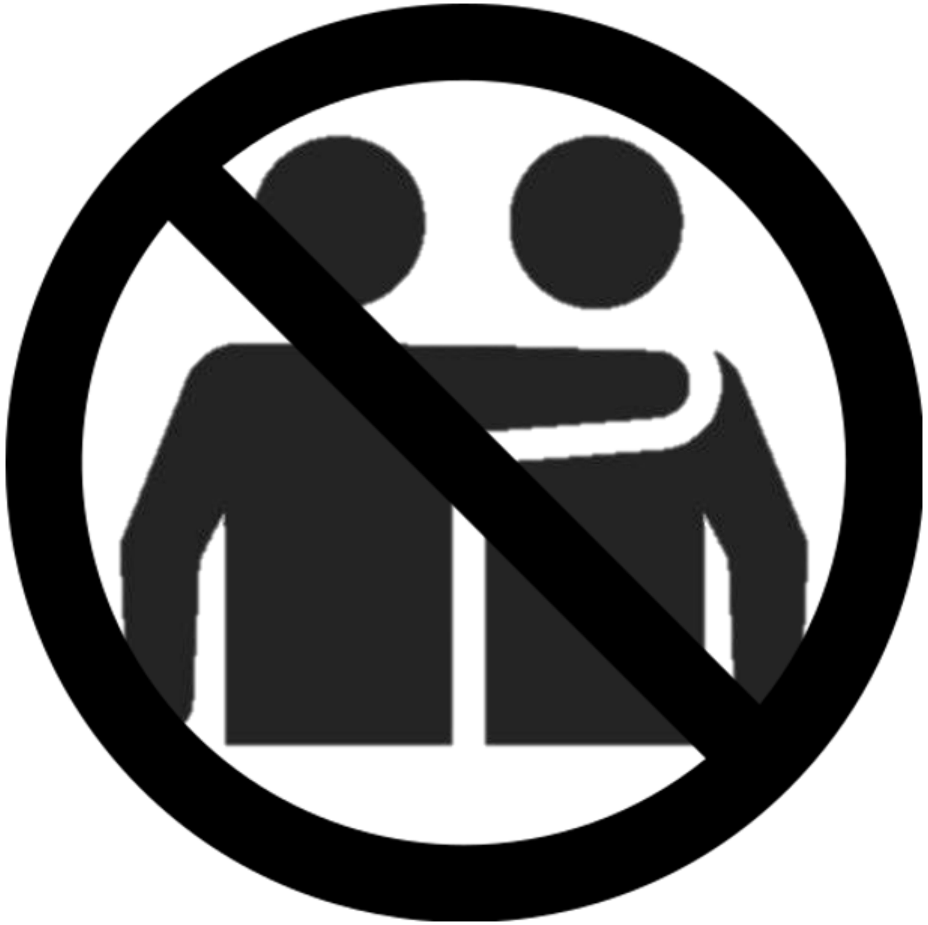

This option means that you will not receive any emotional or social support during your TB treatment.

Physical support during treatment

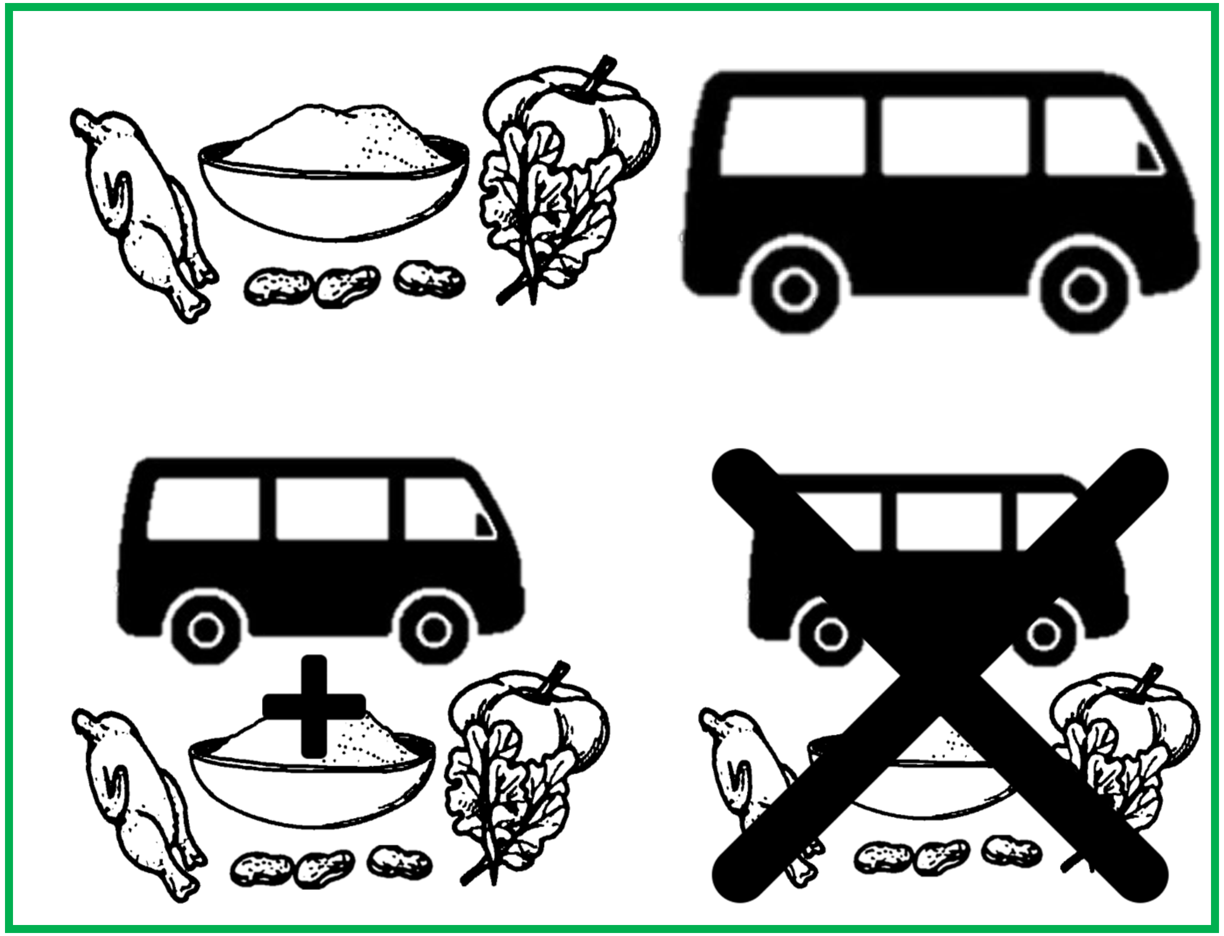

- Sometimes things like not having enough food, or the cost of getting to the health facility can make it difficult to take TB medicine correctly and consistently. Thus, types of physical support, like providing food supplements, or reimbursing the costs of travel to the health facility for visits can be very helpful.

- We will show you **4 different options** for physical support you can receive during TB treatment.

Food supplements each month

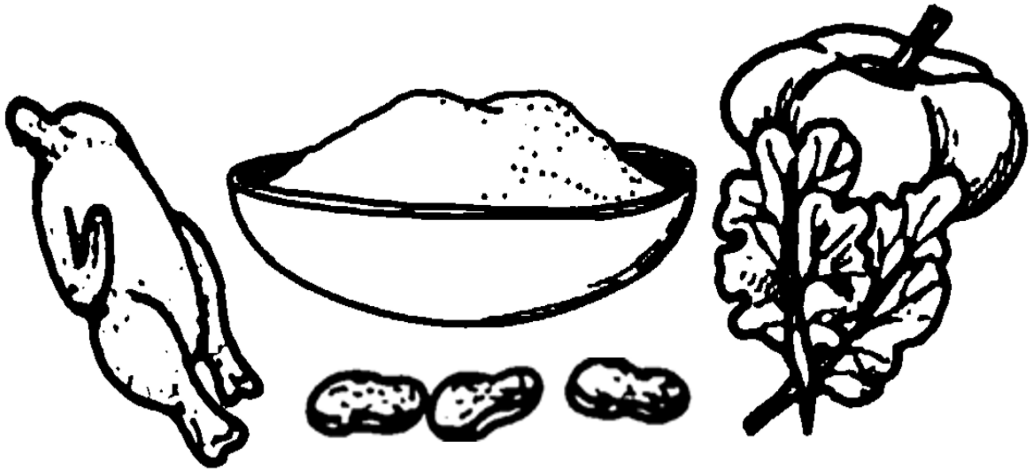

- This option means that every month, you will receive food supplements to help ensure that you and your family have nutritious meals while you are undergoing TB treatment.
- This may include items such as bags of mealie meal and groundnuts, powdered milk and fresh vegetables.

Transport reimbursement for each health facility visit

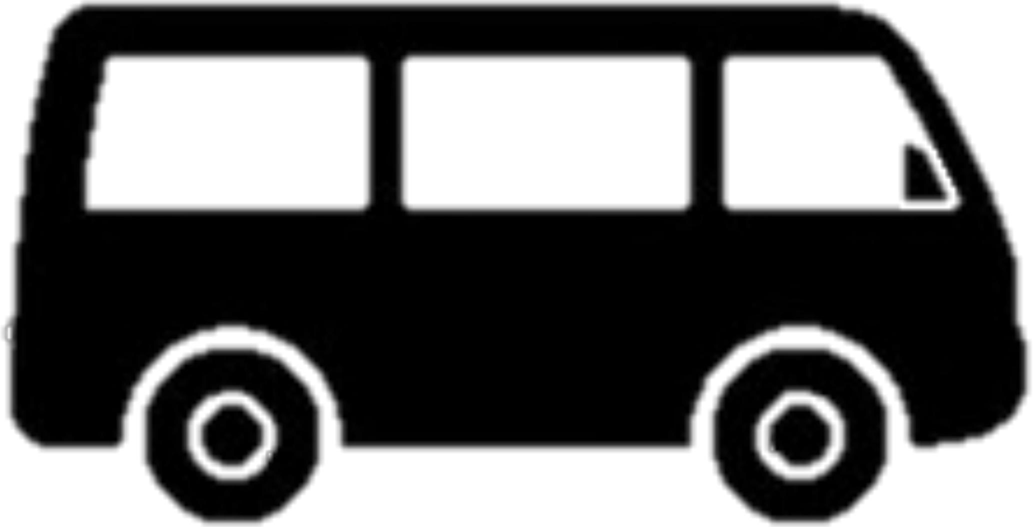

- This option means that each time you visit the health facility, you will enough kwacha to cover your travel costs, making it easier for you to get the care you need.

Food supplements <u>and</u> transport reimbursement

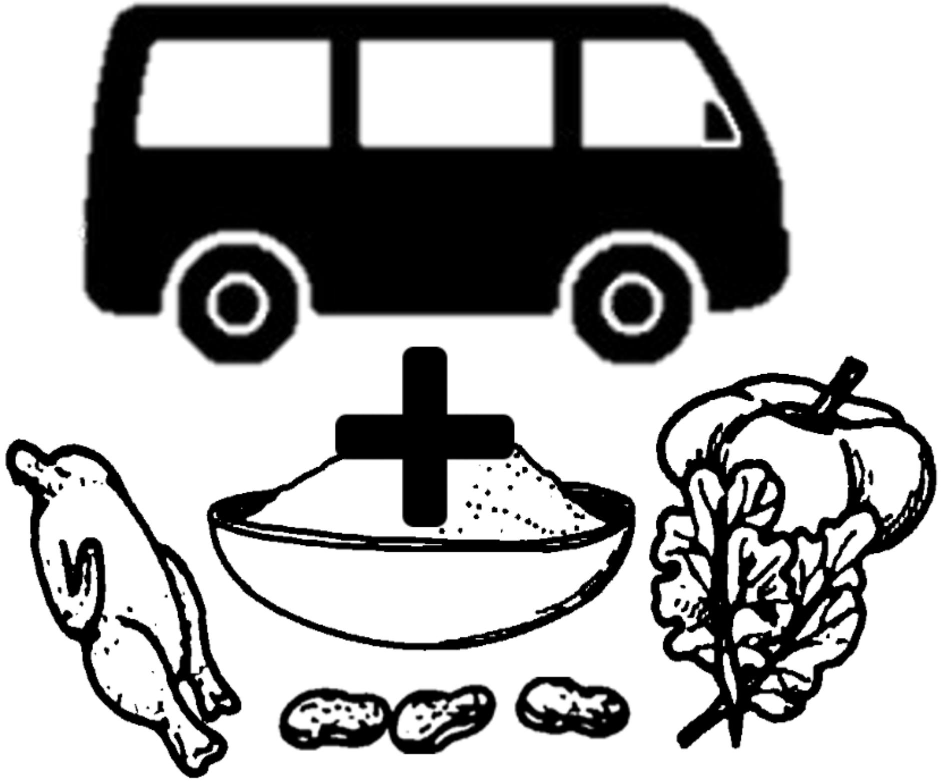

- This option means that every month, you will receive food supplements to help ensure that you and your family have nutritious meals while you are undergoing TB treatment.
- Each time you visit the health facility, you will **ALSO** enough kwacha to cover your travel costs, making it easier for you to get the care you need.

No physical support during treatment

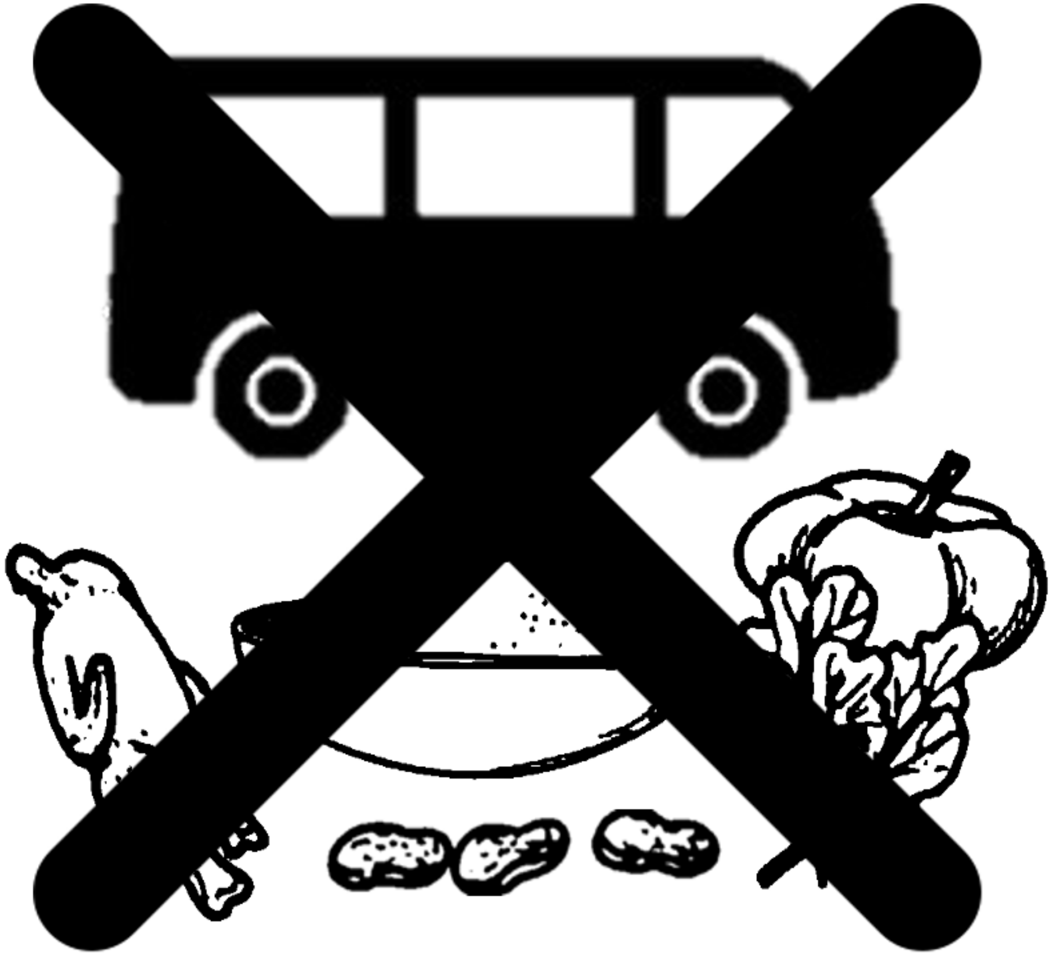

- This option means that you will not receive any form or physical support or help during your TB treatment.

Supplementary File 2. Example choice task

Of the two options below, please pick the TB treatment support package that you think would best meet your needs and that you would prefer.

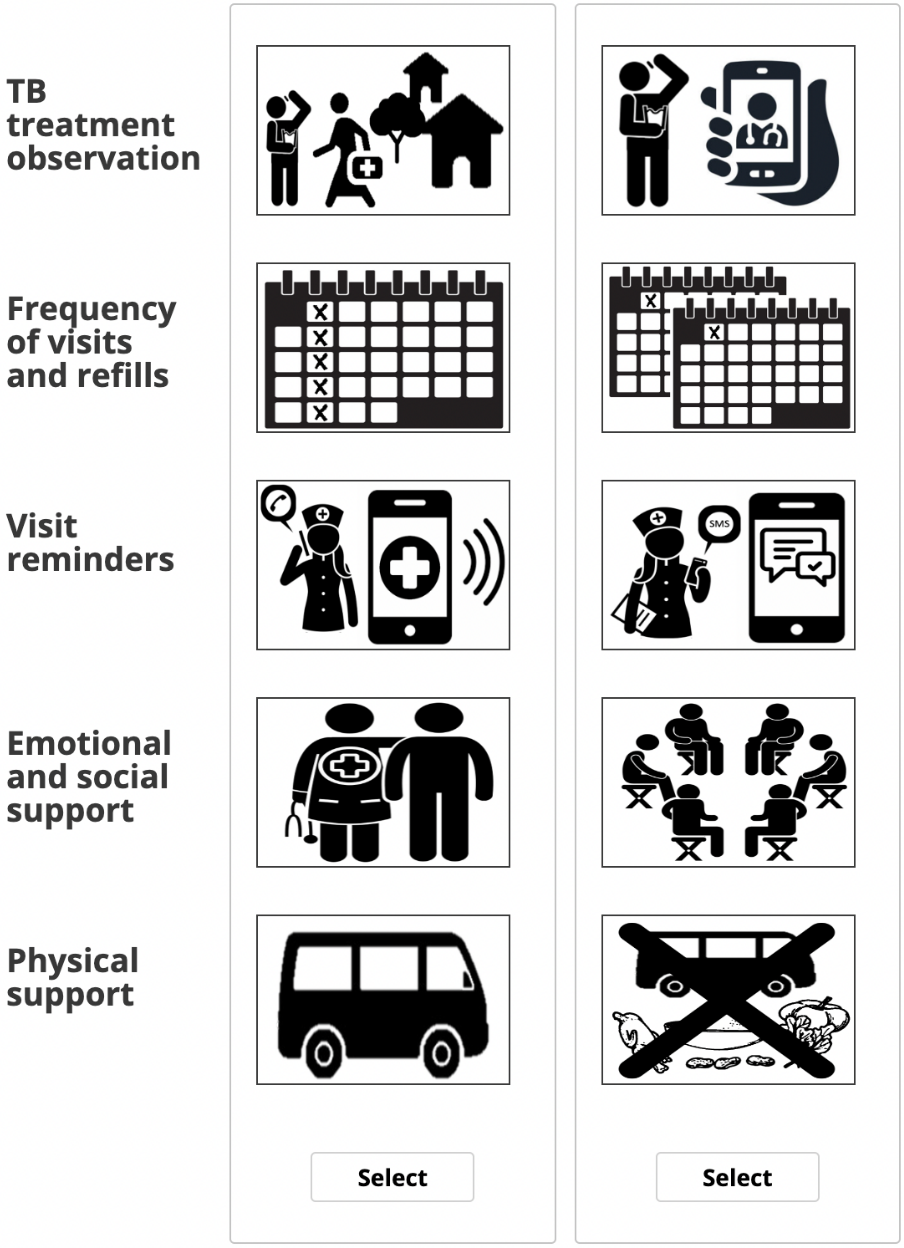

Supplementary File 3. Description of Latent Class Analysis Procedures for Identifying Distinct Preference Groups

For the final model, the number of distinct preference groups was selected by considering which solution optimized statistical fit and the interpretability of preference archetypes represented by each group (see the Table below). Two, three, four, and five-group latent class solutions were evaluated. Since there is no consensus as to which single statistical criterion indicates the best model fit, we used several information criteria to evaluate each latent class solution, including Akaike information criterion (AIC), Bayesian information criterion (BIC), and Consistent Akaike information criterion (CAIC), where lower values indicate a better fit. We also accounted for log-likelihood, minimum class size, and average maximum membership probability.

We first evaluated the preference weights for each attribute level across all solutions to assess the interpretability of the different preference archetypes. The four-group and five-group solutions resulted in the smallest class being inadequate in size (18% and 4%, respectively), with class-specific preferences that were not clearly interpretable or distinct. We therefore focused on two- and three-group solutions, both of which had adequate minimum class sizes and clearly interpretable preference archetypes. Despite a slightly larger BIC and modest decrease in average maximum membership probability (0.89 vs. 0.92), the three-group solution was selected based on improvements in AIC, CAIC, and log-likelihood over the two-group solution, and because the third class provided additional programmatically relevant insight that was obscured in the two-group solution.

Groups	Smallest Group Size	AIC	BIC	CAIC	Log-likelihood	Average maximum membership probability	All groups clearly interpretable and distinct
**2**	34 (34%)	903	1040	1067	-425	0.92	Yes
**3**	29 (29%)	885	1094	1135	-402	0.89	Yes
**4**	18 (18%)	832	1112	1167	-361	0.95	No
**5**	4 (4%)	829	1179	1248	-345	0.93	No
